# Lifestyle-related factors in late midlife as predictors of frailty from late midlife into old age: a longitudinal birth cohort study

**DOI:** 10.1093/ageing/afae066

**Published:** 2024-04-01

**Authors:** Markus J Haapanen, Tuija M Mikkola, Juulia Jylhävä, Niko S Wasenius, Eero Kajantie, Johan G Eriksson, Mikaela B von Bonsdorff

**Affiliations:** Public Health Research Program, Folkhälsan Research Center, Helsinki, Finland; Department of General Practice and Primary Health Care, University of Helsinki, Helsinki, Finland; Department of Medical Epidemiology and Biostatistics, Karolinska Institutet, Stockholm, Sweden; Public Health Research Program, Folkhälsan Research Center, Helsinki, Finland; Public Health Unit, Finnish Institute for Health and Welfare, Helsinki and Oulu, Finland; Clinicum, Faculty of Medicine, University of Helsinki, Helsinki, Finland; Department of Medical Epidemiology and Biostatistics, Karolinska Institutet, Stockholm, Sweden; Faculty of Social Sciences (Health Sciences) and Gerontology Research Center, Tampere University, Tampere, Finland; Public Health Research Program, Folkhälsan Research Center, Helsinki, Finland; Department of General Practice and Primary Health Care, University of Helsinki, Helsinki, Finland; Public Health Unit, Finnish Institute for Health and Welfare, Helsinki and Oulu, Finland; Clinical Medicine Research Unit, Oulu University Hospital and University of Oulu, Oulu, Finland; Department of Clinical and Molecular Medicine, Norwegian University of Science and Technology, Trondheim, Norway; Children’s Hospital, Helsinki University Hospital, Helsinki, Finland; Public Health Research Program, Folkhälsan Research Center, Helsinki, Finland; Department of General Practice and Primary Health Care, University of Helsinki, Helsinki, Finland; Yong Loo Lin School of Medicine, Department of Obstetrics and Gynecology and Human Potential Translational Research Programme, National University Singapore, Singapore, Singapore; Singapore Institute for Clinical Sciences (SICS), Agency for Science, Technology and Research (A*STAR), Brenner Centre for Molecular Medicine, Singapore; Public Health Research Program, Folkhälsan Research Center, Helsinki, Finland; Gerontology Research Center and Faculty of Sport and Health Sciences, University of Jyväskylä, Jyväskylä, Finland

**Keywords:** physical activity, sleep, smoking, alcohol consumption, linear mixed models, older people

## Abstract

**Background:**

Few studies have examined longitudinal changes in lifestyle-related factors and frailty.

**Methods:**

We examined the association between individual lifestyle factors (exercise, diet, sleep, alcohol, smoking and body composition), their sum at baseline, their change over the 17-year follow-up and the rate of change in frailty index values using linear mixed models in a cohort of 2,000 participants aged 57–69 years at baseline.

**Results:**

A higher number of healthy lifestyle-related factors at baseline was associated with lower levels of frailty but not with its rate of change from late midlife into old age. Participants who stopped exercising regularly (adjusted β × Time = 0.19, 95%CI = 0.10, 0.27) and who began experiencing sleeping difficulties (adjusted β × Time = 0.20, 95%CI = 0.10, 0.31) experienced more rapid increases in frailty from late midlife into old age. Conversely, those whose sleep improved (adjusted β × Time = −0.10, 95%CI = −0.23, −0.01) showed a slower increase in frailty from late midlife onwards. Participants letting go of lifestyle-related factors (decline by 3+ factors vs. no change) became more frail faster from late midlife into old age (adjusted β × Time = 0.16, 95% CI = 0.01, 0.30).

**Conclusions:**

Lifestyle-related differences in frailty were already evident in late midlife and persisted into old age. Adopting one new healthy lifestyle-related factor had a small impact on a slightly less steeply increasing level of frailty. Maintaining regular exercise and sleeping habits may help prevent more rapid increases in frailty.

## Key Points

- Lifestyle-related differences in frailty were observed in late midlife and they persisted into old age.
- Adopting one new lifestyle-related factor had a small impact on a slower increase in frailty into old age.
- Changes in regular exercise and sleeping habits were most strongly associated with the development of frailty.
- Cautionary interpretation of findings is warranted due to possible reverse causality and selective survival.

## Introduction

Frailty, characterised by increased vulnerability and reduced physiological reserves, poses challenges to older individuals, increasing the risk of adverse health outcomes, functional decline and increased healthcare utilisation [1]. Understanding the determinants that contribute to the development and progression of frailty become crucial in developing effective preventive strategies and promoting healthy ageing.

Although the origins of frailty can be tracked to earlier life phases [2, 3], a healthier lifestyle in late adulthood and old age has been shown to decrease the risk of frailty [4–13]. The risk of frailty was lower in individuals who adhered to a single healthy lifestyle factor (e.g. relating to smoking, alcohol use, diet, physical activity, sleep and body composition) [4–6, 8, 10–13]. Studies on the total number of healthy lifestyle factors show a decreasing risk of frailty with an increasing number of adhered factors [7–9]. Despite the modifiability of lifestyle factors, few studies have investigated changes in lifestyle and frailty. The study by Gil-Salcedo et al. found a lower hazard of developing physical frailty over 20 years for participants improving or maintaining a higher number of healthy factors over a decade, compared to those consistently following a less healthy lifestyle [8].

This study aimed to explore whether individuals with a less healthy lifestyle would become frailer with older age than those following a healthier lifestyle. Second, we studied the impact of changes in individual lifestyle factors or the total number of lifestyle factors on frailty across older age. Frailty was conceptualised as a frailty index (FI) to better capture subtle temporal changes [14, 15]. Participants from the Helsinki Birth Cohort Study (HBCS) were clinically evaluated for frailty and lifestyle in late midlife (ages 57 to 69 years) and followed for 17 years into old age.

## Materials and methods

### Study design

**Figure 1:**
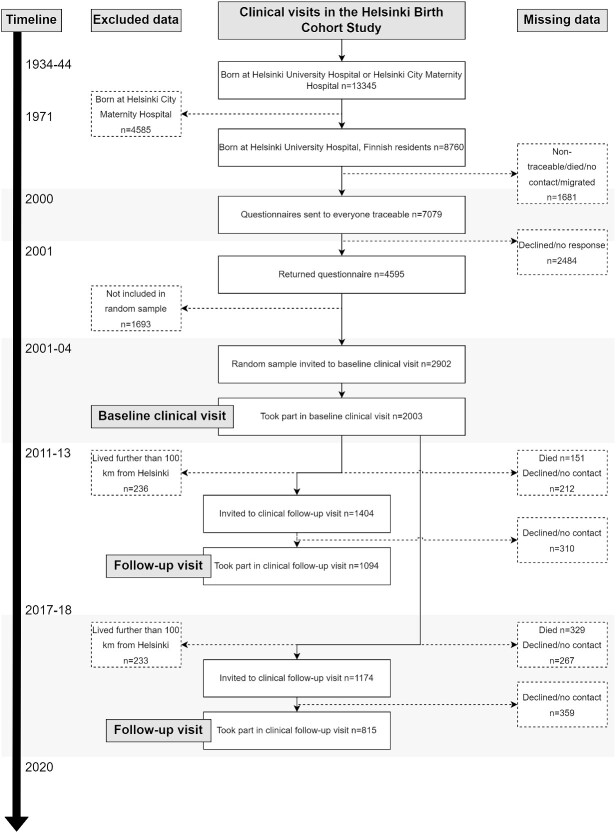
The flow of participants in the Helsinki Birth Cohort Study [3].

This study focuses on HBCS participants (*n* = 13,345) born in Helsinki University Central Hospital (*n* = 8,760) between 1934 and 1944, who visited child welfare clinics in the city and were residents of Finland in 1971 when unique identification numbers were assigned to all Finnish residents [16]. Figure 1 presents the inclusion of participants. A randomly selected sample underwent baseline clinical examinations during 2001–04, with follow-up visits in 2011–13 and 2017–18. Six healthy lifestyle factors (exercise, diet, sleep, smoking, alcohol and body composition) and frailty defined using a 37-item FI [3] were measured at all three visits, except diet, which was measured in 2001–04 and 2011–13. Lifestyle at baseline and its change over the study were used to predict the rate of increase in frailty across older age among 2,000 individuals.

### Healthy lifestyle factors

We focused on six healthy lifestyle factors: regular exercise, adherence to a health-promoting diet, perceived sleep quality, not smoking, infrequent alcohol consumption and maintaining a healthy body composition. Firstly, regular exercise was defined as participating in at least 12.5 metabolic equivalent hours of leisure- time physical activity (LTPA), assessed with the Kuopio Ischemic Heart Disease Risk Factor Study Questionnaire [17]. The cut-off was chosen to align with the minimum LTPA recommendation by the World Health Organization 2020 guidelines [18]. Secondly, participants with Alternative Healthy Eating Index (AHEI) scores of 65 or higher were considered to have a health-promoting diet based on previous observations of lower risk of chronic diseases [19]. Thirdly, participants who indicated ‘I have not experienced any change in my sleeping’ in the Beck Depression Inventory question on changes in sleeping pattern [20] were classified as not having disturbances sleeping. Fourth, non-smokers included participants who had quit or never smoked. Information on pack-years of smoking were available for 812 participants at the 2017–18 follow-up visit. Fifth, participants who reported alcohol consumption frequency of less than once a month, or never, were classified as infrequent consumers. Finally, we defined a healthy body composition as having a percent body fat <25% among men and <35% among women [21]. We preferred the percent body fat over body mass index since it contains information on the proportion of fat tissue and may more accurately detect obesity among older adults. All six healthy lifestyle factors, coded as yes = 1 and no = 0, were summed up to create the main predictor in this study. Changes in individual factors and their sum were calculated by subtracting the baseline measurement from the latest measurement available.

### Frailty

The previously described 41-item FI [3] includes diseases, clinical measurements, laboratory test values, functioning measures and general health information. The FI was created following the standard procedure [22]. Deficit candidates that showed early saturation, had a prevalence of less than 1% or had more than 10% missing data at any of the three measurement occasions were excluded. For this study, we excluded four deficits (*body mass index*, *waist-to-hip ratio*, *physical activity* and *sleep disturbance*) as they were among the studied predictors. Supplementary Table 1 presents the resulting 37 variables and their scoring into deficits. The FI could be calculated for 99.6, 99.9 and 98.5% of participants participating at the three visits. The FI level of ≥0.25 was used to indicate the ‘frail’ state [23].

### Covariates

Childhood socioeconomic status (SES) was categorised into manual workers, lower-middle class, and upper-middle class based on the highest occupational status of the father. Adult SES was assessed by grouping occupational status according to the classification provided by Statistics Finland [24]. Marital status was coded as not married, married, divorced and widowed.

### Statistical methods

The aim of our analysis was to explore the association between individual healthy lifestyle factors, their sum at baseline, their change during the study, and the level of frailty at baseline in late midlife and the rate of change in frailty across increasing age spanning late midlife into old age. To investigate this, we employed linear mixed models with the continuous FI as the outcome and age centred at 57 years, the minimum value in our dataset. We did not find evidence of sex interactions or nonlinear relationships between our predictor variables and frailty.

Age was used as the underlying time scale in all analyses and was therefore inherently adjusted for in the corresponding analyses. Separate models were fitted to assess the associations between the individual lifestyle factors and frailty and the sum of the lifestyle factors and frailty. The fully adjusted model of lifestyle factors at baseline included all six lifestyle factors and was adjusted for sex, marital status, childhood and adult SES. The model based on the sum of lifestyle factors at baseline was adjusted for sex, marital status, childhood and adult SES.

Similarly, we also studied the associations of the changes in the individual lifestyle factors and their sum across older age with frailty in two separate models. The fully adjusted model included the changes in all six individual lifestyle factors and was adjusted for sex, marital status, childhood and adult SES. The analysis of the change in the number (sum) of lifestyle factors was adjusted for sex, marital status, childhood and adult SES. The changes in lifestyle factors were approximations of the change between the baseline and follow-up measurement occasions depending on the data availability of the individual variables and thus not directly interpretable as time-lagged effects.

We also studied the AHEI questionnaire score, MET-hours of LTPA, percent body fat and pack-years of smoking in separate linear mixed models to further understand the associations between continuous scales of diet, physical activity, adiposity, smoking and frailty. The models were adjusted with sex, marital status, childhood and adult SES.

We present unstandardised β estimates of the level of frailty at age 57 years and unstandardised β × Time estimates of the annual rate of change in frailty from late midlife into old age. To improve their interpretability, we multiplied these estimates by 100 and treated them as percentages. Estimates of the level of frailty represent percent higher/lower frailty in late midlife and estimates of the rate of change represent a faster or slower percentage point change in frailty per year compared to the average annual change from midlife to old age. A *P*-value < 0.05 was used as the threshold for statistical significance. We performed the analyses with the R software [25] packages lme4 [26] and lmerTest [27].

## Results

### General characteristics

At baseline, more than a quarter (27.4%) were frail at a mean age of 61.5 years (Table 1). Many came from a lower middle-class background (42.9%) and were married (76.5%). Most participants had either four (30.4%) or three (30.7%) out of the six healthy lifestyle factors. Over the follow-up, the number of healthy lifestyle factors decreased among more than half of the cohort (55.4%), stayed the same among nearly a quarter (23.9%), with one in five (20.7%) adopting new ones. Participants with frailty or fewer healthy lifestyle factors at baseline were less likely to participate in the first clinical follow-up visit (Supplementary Table 2).

**Table 1:** Cohort characteristics

	*N*	Total population (*n* = 2,003)	Women (*n* = 1,075)	Men (*n* = 928)
		Mean (SD)	Mean (SD)	Mean (SD)
**Participant characteristics at baseline**				
Age (years)	2,003	61.5 (2.9)	61.5 (3.0)	61.5 (2.8)
Marital status, *N* (%)	1,978			
Married		1,513 (76.5)	723 (68.2)	790 (86.1)
Not married		136 (6.9)	85 (8.0	51 (5.6)
Divorced		204 (10.3)	152 (14.3)	52 (5.7)
Widowed		125 (6.3)	100 (9.4)	25 (2.7)
Childhood socioeconomic status, *N* (%)	1,989			
Upper middle class		343 (17.2)	165 (15.5)	178 (19.3)
Lower middle class		453 (22.8)	236 (22.1)	217 (23.6)
Labourers		1,193 (60.0)	667 (62.4)	526 (57.1)
Adult socioeconomic status, *N* (%)	2,002			
Upper middle class		286 (14.3)	104 (9.7)	182 (19.6)
Lower middle class		858 (42.9)	603 (56.2)	255 (27.5)
Self-employed		187 (9.3)	91 (8.5)	96 (10.3)
Labourers		671 (33.5)	276 (25.7)	395 (42.6)
Diabetes, *N* (%)	1,998	147 (7.4)	64 (6.0)	83 (9.0)
Hypertension, *N* (%)	1,998	705 (35.3)	370 (34.5)	335 (36.2)
Obesity, *N* (%)	2,001	500 (25.0)	293 (27.3)	207 (22.3)
**Healthy lifestyle factors at baseline**				
Regular exercise, *N* (%)	1,967	1,703 (86.6)	933 (88.0)	770 (84.9)
Health-promoting diet, *N* (%)	1,981	781 (39.4)	493 (46.4)	288 (31.4)
No sleep disturbance, *N* (%)	1,992	1,005 (50.5)	502 (47.2)	503 (54.2)
Does not smoke, *N* (%)	1,987	1,512 (76.1)	844 (79.3)	668 (72.5)
Infrequent drinker, *N* (%)	1,991	405 (20.3)	308 (28.8)	97 (10.5)
Healthy body composition, *N* (%)	1,918	1,118 (58.3)	572 (55.4)	546 (61.6)
Total healthy lifestyle, continuous	1,838	3.33 (1.15)	3.46 (1.14)	3.18 (1.14)
Total healthy lifestyle, *N* (%)	1,838			
5–6		282 (15.4)	182 (18.4)	100 (11.8)
4		559 (30.4)	311 (31.5)	248 (29.1)
3		565 (30.7)	290 (29.4)	275 (32.3)
2		335 (18.2)	165 (16.7)	170 (20.0)
0–1		97 (5.3)	39 (4.0)	58 (6.8)
**Change in lifestyle factors from baseline to follow-up, *N* (%)**				
Improved by 1 ≤ factor	967	200 (20.7)	104 (24.3)	96 (17.8)
No change	967	231 (23.9)	114 (26.7)	117 (21.7)
Declined by 1 factor	967	282 (29.1)	110 (25.8)	172 (31.8)
Declined by 2 factor	967	171 (17.7)	75 (17.6)	96 (17.8)
Declined by 3 ≤ factor	967	83 (8.6)	24 (5.6)	59 (10.9)
**Frailty index**				
Baseline, years 2001–04	1,995	0.20 (0.10)	0.19 (0.10)	0.21 (0.10)
*N* (%) with FI ≥ 0.25		546 (27.4)	329 (30.8)	217 (23.4)
Follow-up, years 2011–13	1,081	0.20 (0.10)	0.22 (0.10)	0.18 (0.09)
*N* (%) with FI ≥ 0.25		311 (28.8)	210 (34.5)	101 (21.4)
Follow-up, years 2017–18	801	0.22 (0.11)	0.23 (0.11)	0.20 (0.10)
*N* (%) with FI ≥ 0.25		265 (33.1)	170 (38.0)	95 (26.8)

Note. SD = standard deviation; FI = frailty index.

### Healthy lifestyle factors and frailty in late midlife

Frail participants had a poorer adherence to five out of six healthy lifestyle factors at baseline, the exception being the proportion of infrequent drinkers (26.7 and 17.9% among frail and not frail participants, respectively; Supplementary Table 3). The level of frailty at baseline was between one and five percent lower for all healthy lifestyle factors except for infrequent drinkers, who showed no association after adjustment for covariates (Table 2). The level of frailty was particularly low among participants with a healthy body composition (adjusted β = −5.40, 95% CI = −6.46, −4.27) or without sleep disturbance (adjusted β = −4.71, 95% CI = −5.65, −3.70; Table 2). Individuals adhering to most healthy lifestyle factors (between five and six) had lower levels of frailty (adjusted β = −3.83, 95% CI = −5.95, −1.61), while those with the least (none or one) experienced higher levels of frailty at baseline (adjusted β = 4.66, 95% CI = 2.95, 6.51) when compared to those having three factors.

**Table 2:** Healthy lifestyle factors at baseline in late midlife as predictors of the level and rate of change in frailty from late midlife into old age

		Level of frailty at age 57 years					Rate of change in frailty from late midlife into old age			
		Age-adjusted^a^		Fully adjusted^b^			Age-adjusted^a^		Fully adjusted^b^	
		*N*	β (95% CI)^c^	N	β (95% CI)^c^		*N*	β × Time (95% CI)^c^	N	β × Time (95% CI)^c^
**Healthy lifestyle factors at baseline**										
Regular exercise^d^		1,967	**−3.64 (−5.14, −2.13)**	1,826	**−2.34 (−3.90, −0.78)**		1,967	0.09 (−0.02, 0.19)	1,826	0.08 (−0.03, 0.18)
Health-promoting diet^d^		1,981	**−1.93 (−3.0, −0.87)**	1,826	**−1.36 (−2.49, −0.36)**		1,981	−0.03 (−0.10, 0.03)	1,826	−0.01 (−0.08, 0.06)
No sleep disturbance^d^		1,992	**−5.14 (−6.17, −4.11)**	1,826	**−4.71 (−5.65, −3.70)**		1,992	**0.08 (0.02, 0.15)**	1,826	**0.09 (0.02, 0.16)**
Does not smoke^d^		1,987	**−2.26 (−3.49, −1.04)**	1,826	**−1.21 (−2.46, −0.10)**		1,987	−0.04 (−0.12, 0.05)	1,826	−0.05 (−0.14, 0.04)
Infrequent drinker^d^		1,991	**2.12 (0.81, 3.44)**	1,826	1.11 (−0.40, 2.34)		1,991	−0.00 (−0.09, 0.08)	1,826	−0.02 (−0.11, 0.07)
Healthy body composition^d^		1,918	**−6.60 (−7.64, −5.57)**	1,826	**−5.40 (−6.46, −4.27)**		1,918	−0.03 (−0.10, 0.04)	1,826	−0.06 (−0.13, 0.02)
**The sum of healthy lifestyle factors at baseline**										
Per increase of one healthy lifestyle factor^e^		1,838	**−2.61 (−3.06, −2.16)**	1,826	**−2.65 (−3.10, −2.21)**		1,838	−0.01 (−0.03, 0.03)	1,826	−0.01 (−0.03, 0.03)
Categories of total healthy lifestyle^e^		1,838		1,826			1,838		1,826	
5–6		282	**−2.99 (−5.09, −0.89)**	281	**−3.83 (−5.95, −1.61)**		282	0.06 (−0.07, 0.19)	281	0.06 (−0.08, 0.18)
4		559	−1.25 (−2.66, 0.16)	553	**−1.69 (−2.98, −0.30)**		559	−0.01 (−0.10, 0.07)	553	−0.01 (−0.10, 0.07)
3		565	Ref.	563	Ref.		565	Ref.	563	Ref.
2		335	**3.16 (1.83, 4.49)**	334	**3.03 (1.61, 4.47)**		335	−0.05 (−0.09, 0.02)	334	−0.01 (−0.09, 0.08)
0–1		97	**4.51 (2.66, 6.36)**	95	**4.66 (2.95, 6.51)**		97	0.07 (−0.06, 0.21)	95	0.06 (−0.08, 0.19)

Note. CI = confidence interval.

^a^Age was used as the underlying time scale and was therefore inherently adjusted for.

^b^Adjusted for sex, childhood and adult socioeconomic status, and marital status. Age was used as the underlying time scale and was therefore inherently adjusted for.

^c^Point estimates refer to the change in FI × 100 units, which translates to percent higher level of frailty at age 57 years and percentage point slower/faster annual increase in FI levels from late midlife into old age.

^d^Analysed separately in age-adjusted models. The fully adjusted model was additionally adjusted for all other healthy lifestyle factors at baseline.

^e^Analysed separately in age-adjusted and fully adjusted models.

### Healthy lifestyle factors in late midlife and frailty from late midlife into old age

Overall, single healthy lifestyle factors or their number at baseline were not associated with the rate of increase in frailty from late midlife into old age (Table 2). However, participants reporting no sleep disturbance experienced a 0.09 percentage point faster annual increase (adjusted β × Time = 0.09, 95% CI = 0.02, 0.16) in their levels of frailty.

### Change in healthy lifestyle factors and frailty from late midlife into old age

**Figure 2:**
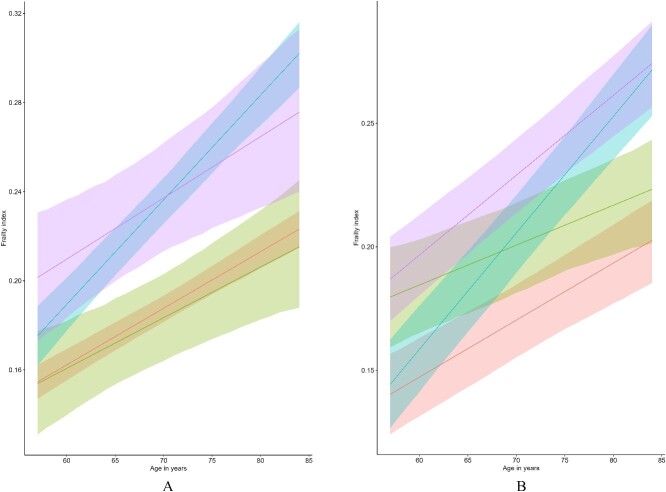
The development of frailty according to change in exercise (left, A) and sleep (right, B) healthy lifestyle factors. The red lines represent groups who consistently exercised or did not report disturbances sleeping. The green lines represent groups who initially did not exercise or reported disturbances sleeping but who then started exercising or whose sleep disturbances discontinued. The blue lines illustrate the levels of frailty among participants who stopped exercising regularly or started experiencing sleeping difficulties. The purple lines represent groups who consistently did not exercise or who consistently reported disturbances sleeping. Figures were adjusted for sex, childhood and adult socioeconomic status, marital status and change in all other lifestyle factors. 95% CI were computed using parametric bootstrapping.

Table 3 and Figure 2 show that compared to participants who kept exercising regularly and did not experience disturbances sleeping, those who stopped regular exercise (adjusted β × Time = 0.19, 95% CI = 0.10, 0.27) and those who began experiencing disturbances sleeping (adjusted β × Time = 0.20, 95% CI = 0.10, 0.31) or consistently had disturbances sleeping (adjusted β × Time = 0.08, 95% CI = 0.00, 0.17) showed more rapid increases in frailty from late midlife into old age. In contrast, participants who reported that they no longer had disturbances sleeping exhibited a slower increase in their levels of frailty (adjusted β × Time = −0.10, 95% CI = −0.23, −0.01). Participants who initially smoked cigarettes but who stopped (adjusted β × Time = 0.12, 95% CI = 0.01, 0.25) or participants whose frequency of alcohol consumption decreased (adjusted β × Time = 0.16, 95% CI = 0.03, 0.31) experienced faster increases in their levels of frailty from late midlife into old age. Compared to participants who maintained lower levels of adiposity, those who consistently had high adiposity became more frail faster from late midlife onwards (adjusted β × Time = 0.11, 95% CI = 0.01, 0.19). Overall, when the change in the sum of the healthy lifestyle factors was studied, for each new healthy lifestyle factor adopted, the rate of frailty increased slightly less steeply (adjusted β × Time = −0.03, 95% CI = −0.06, −0.01) from late midlife to old age.

**Table 3:** Change in healthy lifestyle factors as predictors of the rate of change in frailty from late midlife into old age

	Rate of change in frailty from late midlife into old age			
	Age-adjusted^a^		Fully adjusted^b^	
	*N*	β × Time (95% CI)^c^	*N*	β × Time (95% CI)^c^
**Change in individual healthy lifestyle factors from baseline to follow-up**				
Regular exercise^d^	1,085		964	
Kept exercising regularly	725	Ref.	643	Ref.
Started regular exercise	71	−0.03 (−0.16, 0.11)	69	−0.03 (−0.16, 0.11)
Stopped regular exercise	241	**0.22 (0.13, 0.29)**	212	**0.19 (0.10, 0.27)**
Did not exercise regularly	48	0.02 (−0.13, 0.18)	40	0.03 (−0.15, 0.19)
Health-promoting diet^d^	1,061		964	
Kept eating healthily	336	Ref.	307	Ref.
Started eating healthily	245	**0.12 (0.03, 0.21)**	227	0.07 (−0.03, 0.16)
Stopped eating healthily	135	0.08 (−0.02, 0.19)	119	0.04 (−0.08, 0.15)
Did not eat healthily	345	0.05 (−0.02, 0.13)	311	−0.01 (−0.11, 0.08)
No sleep disturbance^d^	1,109		964	
Consistently without disturbance	321	Ref.	280	Ref.
Lost sleep disturbance	176	−0.07 (−0.17, 0.03)	154	**−0.10 (−0.23, −0.01)**
Gained sleep disturbance	234	**0.25 (0.16, 0.34)**	204	**0.20 (0.10, 0.31)**
Consistently with disturbance	378	**0.09 (0.01, 0.16)**	326	**0.08 (0.00, 0.17)**
Does not smoke^d^	1,111		964	
Did not smoke	674	Ref.	597	Ref.
Quit smoking	133	0.10 (−0.01, 0.21)	109	**0.12 (0.01, 0.25)**
Started smoking	221	0.01 (−0.11, 0.13)	188	0.02 (−0.08, 0.16)
Persistent smoker	83	0.04 (−0.09, 0.18)	70	0.09 (−0.07, 0.24)
Infrequent drinker^d^	1,105		964	
Consistently infrequent	134	Ref.	115	Ref.
Stopped drinking frequently	117	**0.17 (0.03, 0.29)**	98	**0.16 (0.03, 0.31)**
Started drinking more frequently	62	0.16 (−0.03, 0.33)	53	0.14 (−0.05, 0.32)
Consistently frequent drinker	792	0.01 (−0.09, 0.12)	698	0.04 (−0.07, 0.16)
Healthy body composition^d^	1,073		964	
Maintained lower adiposity	458	Ref.	414	Ref.
Adiposity decreased	28	0.02 (−0.19, 0.23)	25	0.00 (−0.22, 0.24)
Adiposity increased	226	**0.09 (0.01, 0.17)**	204	0.06 (−0.02, 0.14)
Maintained high adiposity	361	**0.13 (0.05, 0.21)**	321	**0.11 (0.01, 0.19)**
**Change in total healthy lifestyle from baseline to follow-up**				
Total change, continuous	981	**−0.04 (−0.06, 0.01)**	964	**−0.03 (−0.06, −0.01)**
Total change	981		964	
Improved by 1 ≤ factors	281	0.00 (−0.10, 0.10)	272	0.01 (−0.01, 0.11)
No change	304	Ref.	289	Ref.
Declined by 1 factor	247	−0.04 (−0.14, 0.05)	236	−0.04 (−0.13, 0.06)
Declined by 2 factors	109	0.08 (−0.02, 0.19)	108	0.11 (−0.00, 0.23)
Declined by 3 ≤ factors	40	**0.15 (0.01, 0.30)**	38	**0.16 (0.01, 0.30)**

Note. CI = confidence interval.

^a^Age was used as the underlying time scale and was therefore inherently adjusted for.

^b^Adjusted for sex, childhood and adult socioeconomic status, and marital status. Age was used as the underlying time scale and was therefore inherently adjusted for.

^c^Point estimates refer to the change in FI × 100 units, which translates to percentage point slower/faster annual increase in FI levels from late midlife into old age.

^d^Analysed separately in age-adjusted models. The fully adjusted model was additionally adjusted for change in all other healthy lifestyle factors.

### Continuous lifestyle factors, baseline frailty and rate of change in frailty into old age

Participants engaging in more weekly LTPA or with higher AHEI scores at baseline had lower levels of frailty in late midlife but no association with the rate of change in frailty into old age (Supplementary Table 4). Pack-years of smoking were not associated with frailty at baseline or its rate of change. Participants with a higher percentage body fat at baseline were more frail at baseline and also experienced slightly faster increases in their levels of frailty into old age (Supplementary Table 4).

## Discussion

Previously, greater adherence to healthy lifestyle factors and sustained adherence to multiple factors over ten years were linked to a lower hazard of physical frailty over the following two decades [8]. We aimed to extend these findings by examining deficit accumulation across homeostatic systems using the FI and studying changes in healthy lifestyle factors and frailty over time. We observed differences in frailty associated with lifestyle factors as early as in late midlife. While changes in the specific domains of a healthy lifestyle, such as giving up regular exercise or newly acquired sleep disturbance, were linked to a faster pace at which frailty increased, an increase in the total number of healthy lifestyle factors was only weakly associated with a slightly slower rate of frailty increase from into old age. The findings highlight the importance of adopting a healthy lifestyle from a young age and maintaining regular exercise and sleep routines in late middle age and beyond to prevent frailty.

### Healthy lifestyle factors and frailty

Our study corroborates previous studies showing cross-sectional and temporal associations between a less healthy lifestyle and frailty [4–13]. In line with prior research [7–9], we found an inverse association between the number of healthy lifestyle factors and frailty. Of the six factors studied (exercise, diet, sleep, smoking, alcohol and body composition), all but alcohol showed associations with lower levels of frailty. Notably, sleep and body composition had the strongest associations with prevalent frailty. While limited studies include sleep as part of multiple lifestyle factors and frailty [7], some suggested a higher risk of incident frailty among individuals with poorer sleep [6, 13], while others did not [7]. More studies agree on an association between body anthropometry and frailty [8, 10–12], with further evidence suggesting that greater adiposity could further accelerate deficit accumulation [28]. The association between alcohol consumption and frailty is less clear, with evidence suggesting an association between higher alcohol intake and lower risk of frailty [5, 9].

### Healthy lifestyle factors and changes in frailty

Although there were initial differences in frailty based on lifestyle factors in late midlife, the rate at which frailty increased thereafter was similar for participants with and without healthy lifestyle factors. Importantly, there was no evidence that having fewer healthy lifestyle factors led to a widening gap in frailty with older age. Initial differences in frailty observed in late midlife persisted into old age, except for perceived sleep disturbances. Those reporting no sleep disturbances had slightly faster increases in frailty, but the impact was relatively small compared to the significantly lower levels of frailty observed in late midlife for this group.

### Change in healthy lifestyle factors and changes in frailty

We investigated how changes in healthy lifestyle factors and their number could relate to the rate of change in frailty from late midlife into old age. Our findings suggest that adding one healthy lifestyle factor had a statistically significant but minimal association with a slightly slower increase in frailty into old age. Extending prior findings, we examined changes in individual lifestyle factors. Participants who stopped adhering to minimum physical activity recommendations or experienced new sleep disturbances had faster increases in frailty into old age. This emphasises the importance of physical activity and sleep in preventing frailty. Conversely, those who initially experienced sleep disturbances but which resolved during the study showed slower increases in frailty into old age, aligning with an earlier study [13] showing improved frailty status in participants with healthy sleep duration and no snoring.

Participants who stopped smoking or decreased their frequency of alcohol consumption exhibited faster increases in their levels of frailty from midlife into old age. The reasons behind these changes remain unclear, but it is possible that lifestyle improvements were driven by health-related factors, such as newly diagnosed health problems. While pack-years of smoking or consistent smoking were not associated with frailty in our study, the results were limited to data on participants who had survived into old age.

### Implications

We found lifestyle-attributable differences in frailty in late midlife that persisted without a widening gap into old age. This implies that lifestyle-related changes in frailty were already present by late midlife, emphasising the need for a focus on lifestyle changes in younger age groups. Similar increases in frailty were observed among participants with and without healthy lifestyle factors, indicating no evidence of a widening gap in frailty across older age. The association between an increase of one new healthy lifestyle factor and frailty was small but statistically significant, suggesting potential added benefit of adopting a healthier lifestyle in older age. Changes in exercise and sleep emerged as potential factors in preventing future decline in frailty. Improvements in perceived sleep quality were associated with slower increases in frailty, supporting sleep as an important factor in frailty. Future intervention studies on sleep and frailty are needed to study the effects of sleep interventions on frailty.

### Strengths and weaknesses

A cautious interpretation of our findings is warranted due to several limitations. Firstly, the assessment of healthy lifestyle factors relied on self-reported questionnaires, which may introduce recall bias and subjective interpretation. Alcohol use was measured based on frequency alone, lacking information on quantity or risk-level drinking. Sleep was assessed using participants’ self-perceived changes sleeping, allowing individuals with poor sleep quality but no changes sleeping to be scored as healthy. Continuous scales of physical activity, diet and body composition were dichotomised, potentially leading to a loss of information. All six items of the sum score were weighted equally, potentially undermining, or amplifying their significance on frailty. The potential impact of health-related or other reasons on the participants’ gain or abandonment of healthy lifestyle factors remains uncertain. Moreover, participants with few healthy lifestyle factors or frailty were more likely to die or discontinue for other reasons, potentially underestimating our findings due to a healthy survivor effect. Our results, particularly those regarding changes in lifestyle factors, may be influenced by survival bias and reverse causality. Change in some healthy lifestyle factors was rare, limiting our ability to detect associations. Lastly, the generalisability of our findings is limited as our study focused on individuals of Finnish ancestry born in Helsinki between 1934 and 1944.

## Conclusions

In conclusion, lifestyle-attributed differences in frailty were evident in late midlife and persisted into old age. Failure to meet minimum physical activity recommendations or experiencing sleeping difficulties was associated with faster increases in frailty into old age, while reporting improved sleep was associated with slower increases. Adopting one new healthy lifestyle factor had only minimal associations with the participants becoming slightly less frail from late midlife into old age.

## Supplementary Material

aa-23-1723-File005_afae066
