# Supplementary material for: Lifestyle-related factors in late midlife as predictors of frailty from late midlife into old age: a longitudinal birth cohort study
Source: Age Ageing. 2024 Apr 1;53(4):afae066. doi: 10.1093/ageing/afae066 (PMC10982848; doi:10.1093/ageing/afae066)
Supplement: aa-23-1723-File005_afae066 [file aa-23-1723-file005_afae066.docx]

Healthy lifestyle factors in late midlife as predictors of frailty from late midlife into old age: a longitudinal birth cohort study

Supplementary Material

**Supplementary Table 1**. List of the 37 variables included in the frailty index in the Helsinki Birth Cohort Study.

**Supplementary Table 2**. Non-participation at the follow-up visit in 2011-2013 according to healthy

lifestyle factors and frailty at baseline.

**Supplementary Table 3**. Cohort characteristics according to frailty status at baseline.

**Supplementary Table 4**. Continuous measures of healthy lifestyle factors at baseline as predictors of the level and rate of change in frailty from late midlife into old age.

**Supplementary Table 1.** List of the 37 variables included in the frailty index in the Helsinki Birth Cohort Study.

| Variable name | | Scoring |
| --- | --- | --- |
| 1 | Self-reported angina pectoris as diagnosed by a doctor^a^ | Yes=1; no=0 |
| 2 | Self-reported asthma as diagnosed by a doctor^a^, special reimbursement for obstructive airway disease medication^b^ | Yes=1; no=0 |
| 3 | Self-reported cancer as diagnosed by a doctor^a^ | Yes=1; no=0 |
| 4 | Self-reported claudication as diagnosed by a doctor^a^ | Yes=1; no=0 |
| 5 | Special reimbursement for antiarrhythmic medication^b^ | Yes=1; no=0 |
| 6 | Self-reported depression by a doctor^a^ | Yes=1; no=0 |
| 7 | Self-reported diabetes as diagnosed by a doctor^a^, special reimbursement for diabetes medication^b^ | Yes=1; no=0 |
| 8 | Self-reported emphysema as diagnosed by a doctor^a^ | Yes=1; no=0 |
| 9 | Special reimbursement for glaucoma medication^b^ | Yes=1; no=0 |
| 10 | Self-reported heart failure as diagnosed by a doctor^a^ | Yes=1; no=0 |
| 11 | Self-reported hypertension as diagnosed by a doctor^a^, special reimbursement for antihypertensive drugs^b^ | Yes=1; no=0 |
| 12 | Self-reported myocardial infarct as diagnosed by a doctor^a^ | Yes=1; no=0 |
| 13 | Self-reported osteoporosis as diagnosed by a doctor^a^ | Yes=1; no=0 |
| 14 | Self-reported stroke as diagnosed by a doctor^a^ | Yes=1; no=0 |
| 15 | General health^c^ | Poor=1; fair=0.75; good=0.50; very good=0.25; excellent=0 |
| 16 | Health compared to one year ago^c^ | Worse=1; same/better=0 |
| 17 | Health limits vigorous activities^c^ | Yes=1; yes a bit=0.50; no=0 |
| 18 | Health limits moderate activities^c^ | Yes=1; yes a bit=0.50; no=0 |
| 19 | Health limits lifting or carrying groceries^c^ | Yes=1; yes a bit=0.50; no=0 |
| 20 | Health limits climbing several flights of stairs^c^ | Yes=1; yes a bit=0.50; no=0 |
| 21 | Health limits bending, kneeling, or stooping^c^ | Yes=1; yes a bit=0.50; no=0 |
| 22 | Health limits walking more than a kilometre^c^ | Yes=1; yes a bit=0.50; no=0 |
| 23 | Health limits walking more than 100 metres^c^ | Yes=1; yes a bit=0.50; no=0 |
| 24 | Health limits bathing or dressing^c^ | Yes=1; yes a bit=0.50; no=0 |
| 25 | Physical health limits the kind of work or other activities^c^ | Yes=1; no=0 |
| 26 | Bodily pain^c^ | Severe/very severe=1; moderate=0.75; mild=0.50; very mild=0.25; no pain=0 |
| 27 | Feel full of life^c^ | None=1; some/little=0.50; all/most/good bit=0 |
| 28 | Feel tired^c^ | All/most/good bit=1; some/little=0.50; none=0 |
| 29 | Heart rate (bpm) | <60 or >100=1; ≥60 and ≤100=0 |
| 30 | Systolic blood pressure measured to be ≥ 160 mmHg or diastolic blood pressure measured to be ≥ 100 mmHg | Yes=1; no=0 |
| 31 | Abnormal fasting glucose (≥ 6.1 mmol/l)^d^ | Yes=1; no=0 |
| 32 | High total cholesterol level (> 5.0 mmol/l)^d^ | Yes=1; no=0 |
| 33 | Low high-density lipoprotein level (men < 1.00 mmol/l, women < 1.20 mmol/l)^d^ | Yes=1; no=0 |
| 34 | Abnormal alanine amino transferase / aspartate transaminase level (ALT > 50 U/l for men and > 35 U/l for women; AST > 45 U/l for men and > 35 U/l for women)^d^ | Yes=1; no=0 |
| 35 | Less interested in other people than before^e^ | Yes=1; no=0 |
| 36 | Changes in appetite^e^ | Less than usual=1; no change=0 |
| 37 | Weight loss^e^ | 2.5 kg or greater=1; weight has been stable=0 |

Note. ^a^Assessed using questionnaires at clinical baseline and follow-up visits; ^b^prescription medicines reimbursed out of National Health Insurance until the year 2017; ^c^from the RAND-36/SF-36 questionnaire (1); ^d^cut-offs indicating abnormal test results; ^e^from the Beck Depression Inventory (BDI) (2) questionnaire.

1. RAND. The RAND 36-Item Health Survey The RAND 36-Item Health Survey. Heal (San Fr. 1992;2(March):91–7.

2. Beck AT, Steer RA BG. Manual for the Beck Depression Inventory-II. San Antonio TX: Psychological Corporation; 1996.

**Supplementary Table 2.** Non-participation at the follow-up visit in 2011-2013 according to healthy

|  | |  | Invited to clinical follow-up visit | Declined/no contact/lived further away | Died | P |
| --- | --- | --- | --- | --- | --- | --- |
|  | |  | n=1404 | n=448 | n=151 |  |
|  | **Healthy lifestyle factors at baseline** | | | | | |
| Regular exercise, N (%) | |  | 1210 (87.5) | 373 (85.2) | 120 (82.2) | 0.12 |
| Health-promoting diet, N (%) | |  | 589 (42.5) | 156 (35.0) | 36 (24.3) | <0.001 |
| No sleep disturbance, N (%) | |  | 701 (50.3) | 230 (51.3) | 74 (49.7) | 0.9 |
| Does not smoke, N (%) | |  | 1087 (78.0) | 336 (75.7) | 89 (59.3) | <0.001 |
| Infrequent drinker, N (%) | |  | 270 (19.4) | 109 (24.4) | 26 (17.3) | 0.046 |
| Healthy body composition, N (%) | |  | 815 (60.2) | 230 (54.4) | 73 (51.4) | 0.023 |
| Total healthy lifestyle, N (%) | |  | 1297 | 409 | 132 | 0.003 |
| 5-6 | |  | 210 (16.2) | 63 (15.4) | 9 (6.8) |  |
| 4 | |  | 407 (31.4) | 122 (29.8) | 30 (22.7) |  |
| 3 | |  | 401 (30.9) | 117 (28.6) | 47 (35.6) |  |
| 2 | |  | 217 (16.7) | 85 (20.8) | 33 (25.0) |  |
| 0-1 | |  | 62 (4.8) | 22 (5.4) | 13 (9.9) |  |
|  | **Frailty at baseline** | | | | | |
| Frailty index (FI), mean (SD) | |  | 0.19 (0.09) | 0.21 (0.11) | 0.25 (0.12) | <0.001 |
| Frail (FI ≥ 0.25), N (%) | |  | 329 (23.5) | 147 (33.0) | 70 (46.4) | <0.001 |

lifestyle factors and frailty at baseline.

Note. SD=standard deviation.

**Supplementary Table 3.** Cohort characteristics according to frailty status at baseline.

|  | Not frail (n=1428, 71.3%)^a^ | Frail (n=575, 28.7%)^a^ | P |
| --- | --- | --- | --- |
|  | N (%) | N (%) |  |
| **Healthy lifestyle factors at baseline** | | | |
| Regular exercise | 1269 (88.6) | 426 (81.0) | <0.001 |
| Health-promoting diet | 595 (41.4) | 181 (33.8) | 0.002 |
| No sleep disturbance | 818 (56.7) | 185 (34.1) | <0.001 |
| Does not smoke | 1122 (77.9) | 387 (71.3) | 0.002 |
| Infrequent drinker | 258 (17.9) | 145 (26.7) | <0.001 |
| Healthy body composition | 923 (66.2) | 192 (37.3) | <0.001 |
| Total healthy lifestyle | 1332 | 506 | <0.001 |
| 5-6 | 251 (18.9) | 31 (6.1) |  |
| 4 | 455 (34.2) | 104 (20.6) |  |
| 3 | 411 (30.8) | 154 (30.4) |  |
| 2 | 175 (13.1) | 160 (31.6) |  |
| 0-1 | 40 (3.0) | 57 (11.3) |  |
| **Change in individual healthy lifestyle factors from baseline to follow-up** | | |  |
| Regular exercise | 868 | 217 | <0.001 |
| Kept exercising regularly | 613 (70.6) | 112 (51.6) |  |
| Started regular exercise | 56 (6.5) | 15 (6.9) |  |
| Stopped regular exercise | 170 (19.6) | 71 (30.7) |  |
| Did not exercise regularly | 29 (3.3) | 19 (8.8) |  |
| Health-promoting diet | 841 | 220 | 0.7 |
| Kept eating healthily | 273 (32.5) | 63 (28.6) |  |
| Started eating healthily | 190 (22.6) | 55 (25.0) |  |
| Stopped eating healthily | 108 (12.8) | 27 (12.3) |  |
| Did not eat healthily | 270 (32.1) | 75 (34.1) |  |
| No sleep disturbance | 878 | 231 | <0.001 |
| Consistently without disturbance | 292 (33.3) | 29 (12.6) |  |
| Lost sleep disturbance | 130 (14.8) | 46 (19.9) |  |
| Gained sleep disturbance | 202 (23.0) | 32 (13.8) |  |
| Consistently with disturbance | 254 (28.9) | 124 (53.7) |  |
| Does not smoke | 880 | 231 | 0.001 |
| Did not smoke | 559 (63.5) | 115 (49.8) |  |
| Quit smoking | 101 (11.5) | 32 (13.8) |  |
| Started smoking | 157 (17.8) | 64 (27.7) |  |
| Persistent smoker | 63 (7.2) | 20 (8.7) |  |
| Infrequent drinker | 876 | 229 | 0.030 |
| Consistently infrequent | 98 (11.2) | 36 (15.7) |  |
| Stopped drinking frequently | 86 (9.8) | 31 (13.5) |  |
| Started drinking more frequently | 46 (5.3) | 16 (7.0) |  |
| Consistently frequent drinker | 646 (73.7) | 146 (63.8) |  |
| Healthy body composition | 844 | 229 | <0.001 |
| Maintained low adiposity | 394 (46.7) | 64 (27.9) |  |
| Decreasing adiposity | 21 (2.5) | 7 (3.1) |  |
| Increasing adiposity | 197 (23.3) | 29 (12.7) |  |
| Maintained high adiposity | 232 (27.5) | 129 (56.3) |  |
| **Change in lifestyle from baseline to follow-up** | 775 | 192 | 0.5 |
| Improved by 1 ≤ points | 198 (25.6) | 60 (31.3) |  |
| No change | 227 (29.3) | 54 (28.1) |  |
| Declined by 1 point | 214 (27.6) | 45 (23.4) |  |
| Declined by 2 points | 104 (13.4) | 24 (12.5) |  |
| Declined by 3 ≤ points | 32 (4.1) | 9 (4.7) |  |

Note. ^a^Based on frailty index values of < 0.25 and ≥ 0.25

**Supplementary Table 4.** Continuous measures of healthy lifestyle factors at baseline as predictors of the level and rate of change in frailty from late midlife into old age.

|  |  | Level of frailty at age 57 years | |  | Rate of change in frailty from late midlife into old age | |
| --- | --- | --- | --- | --- | --- | --- |
|  |  | Age-adjusted^a^ | Fully-adjusted^b^ |  | Age-adjusted^a^ | Fully-adjusted^b^ |
|  |  | β (95% CI)^c^ | β (95% CI)^c^ |  | β × Time (95% CI)^c^ | β × Time (95% CI)^c^ |
| **Continuous healthy lifestyle factors at baseline^d^** | | | | | | |
| MET hours of weekly LTPA^e^ |  | **-0.016 (-0.029, -0.002)** | **-0.017 (-0.030, -0.004)** |  | 0.001 (-0.002, 0.002) | 0.001 (-0.002, 0.002) |
| AHEI score^f^ |  | **-0.130 (-0.188, -0.073)** | **-0.128 (-0.182, -0.072)** |  | -0.001 (-0.005, 0.003) | -0.001 (-0.004, 0.002) |
| Pack-years of smoking^g^ |  | 0.022 (-0.022, 0.065) | 0.039 (-0.004, 0.082) |  | 0.002 (-0.001, 0.004) | 0.002 (-0.001, 0.004) |
| Percent body fat^h^ |  | **0.401 (0.338, 0.466)** | **0.521 (0.452, 0.584)** |  | **0.001 (0.001, 0.001)** | **0.001 (0.001, 0.001)** |

Note. Abbreviations: CI=confidence interval; MET=metabolic equivalent of task; LTPA=leisure time physical activity; AHEI=alternative healthy eating index.

^a^ Age was used as the underlying time scale and was therefore inherently adjusted for.

^b^ Adjusted for sex, childhood and adult socioeconomic status, and marital status. Age was used as the underlying time scale and was therefore inherently adjusted for.

^c^ Point estimates refer to the change in FI × 100 units, which translate to percent higher level of frailty at age 57 years and percentage point slower/faster annual increase in FI levels from late midlife into old age.

^d^ Variables analysed in separate models.

^e^n=1967. ^f^n=1981. ^g^n=812. ^h^n=1918.
